# Denning habits of free-ranging dogs reveal preference for human proximity

**DOI:** 10.1038/srep32014

**Published:** 2016-08-18

**Authors:** Sreejani Sen Majumder, Manabi Paul, Shubhra Sau, Anindita Bhadra

**Affiliations:** 1 Department of Biological Sciences, Indian Institute of Science Education and Research Kolkata, India

## Abstract

Dens are crucial in the early development of many mammals, making den site selection an important component of parental care in such species. Resource availability and shelter from predators primarily govern den selection. Species inhabiting human-dominated landscapes typically den away from human disturbance, often shifting dens to avoid humans during the early life of their young. Domesticated dogs have evolved in human proximity over centuries, being bred and reared in human homes for generations. While pets rely on their owners for shelter and care, free-ranging dogs roam uncared, and typically whelp in dens. We conducted a study on 148 free-ranging dog dens in India to understand their denning habits. Distance from resources influenced den choice, but anthropogenic disturbance did not. Dens were found in areas of high human activity, and begging from humans was preferred over scavenging. A study on 15 pregnant females revealed that females actively searched for denning sites, rejecting several intermediate ones before selecting the final den. We propose that the obvious preference of dogs for denning close to humans is a behavioural adaptation that helps them to survive in the urban landscape, in spite of the high human induced mortality during the early life of pups.

Parental care is defined as “any form of parental behaviour that appears likely to increase the fitness of offspring”^1^. This broad definition includes a host of behaviours, from allocation of resources for eggs prior to mating and offspring provisioning after birth to nest guarding and suckling^1^,^2^. The most basic, and perhaps common manifestation of parental care is the building or acquisition of nesting or denning sites that can provide protection to the offspring during the early stages of their development^3^,^4^. In mammals, parental care involves suckling which occurs over an extended period of time, and demands high energy investment from the mothers^5^. Carnivorous mammals typically give birth to relatively underdeveloped young that need complete care during their early life for survival^4^,^6^,^7^. In such species, the den plays an important role in the development of the young, providing shelter from the environment, optimal ambient conditions for successful development and protection from predators^8^,^9^,^10^,^11^. Not surprisingly, predator avoidance and proximity to resources have been shown to be two important factors that drive den site selection in many species^8^,^10^,^12^,^13^,^14^,^15^,^16^,^17^,^18^,^19^,^20^,^21^,^22^,^23^,^24^,^25^,^26^.

Rapid urbanization has resulted in fragmentation of habitats available to wildlife worldwide, forcing animals to live in close proximity to humans^27^,^28^. Some animals like skunks (*Spilogale putorius*) in USA and swfit foxes (*Vulpes velox*) in Canada have adapted to this habitat change by using manmade structures as dens^12^,^29^,^30^, placing their dens in close proximity to roads^8^,^12^,^30^ and/or human settlements^12^,^17^,^30^. Some species, however, avoid humans by selecting den sites away from human activity^14^,^18^,^31^. Thus the urban environment provides ample ground for animal-human conflict as well as mutualism, and understanding the denning habits of urban species may contribute to their conservation^21^,^32^,^33^.

Canids are mostly mesocarnivores, often acting as top predators in food chains, have a wide distribution, spanning all continents but Antarctica, and occupy diverse habitats, from undisturbed forests to metropolitan areas^17^,^34^,^35^,^36^,^37^,^38^,^39^,^40^,^41^,^42^,^43^. Some canids like dholes (*Cuon alpinus*) and Arctic foxes (*Vulpes lagopus*) live far from human settlements and rarely interact with humans, but others like gray wolves (*Canis lupus lupus*), coyotes (*Canis latrans*) and dingoes (*Canis dingo*) have adapted to living in and around human habitation^20^,^44^,^45^. The domesticated dog (*Canis familiaris*) has evolved from gray wolves through a process of domestication. While pet dogs are capable of leading a lifestyle completely dependent on humans^46^,^47^,^48^,^49^, free-ranging dogs depend on human-generated waste for their sustenance, but are not under direct human supervision^50^. Nearly 80% of the world’s dog population comprises of free-ranging dogs, and these dogs are the best model systems for understanding the unsolved puzzle of dog domestication^51^,^52^,^53^. Pet dogs breed and whelp under human supervision, but show basic denning behaviours like selecting a preferred spot, digging into the ground and tearing bedding material prior to whelping^54^. They have also been reported to abandon the den suddenly in order to give birth in the proximity of a preferred human^54^. In contrast, wolves tend to den away from human activities, and are intolerant of humans approaching their dens^15^,^31^,^55^,^56^,^57^,^58^,^59^. Coyotes inhabit human dominated environments, but move their pups to new den sites whenever disturbed by humans^60^. Foxes also tend to have birthing dens in man-made structures and human dominated landscapes, but in areas where human disturbance is low, and they often shift dens when disturbed^61^,^62^,^63^. Thus pet dogs seem to have retained the ancestral habit of denning, but have shifted their preference to den close to preferred humans as a result of domestication. Free-ranging dogs could provide us an insight to this transition in denning behaviour, and would contribute to our understanding the various factors that led to the success of dogs in adapting to urban and semi-urban habitats across the globe.

Free-ranging dogs in India are found in all possible human habitations, from forest fringes to metropolitan cities, and they are typically scavengers, surviving on scraps and wastes from human kitchens^41^,^53^,^64^. They breed in and around human habitations, and, unlike their wilder relatives, do not avoid human interactions during pup rearing^5^. Mothers are highly protective, and can get quite aggressive while guarding pups^65^, but shifting of dens as a response to anthropogenic disturbance has not yet been reported in these dogs. On the other hand, there is very high level of human induced mortality in the early life of pups^66^. Thus humans are a potential threat to the pups, and thus the free-ranging dogs in India are a good model system for understanding the choice of denning sites in the face of conflict with humans. In this study we aim to characterize den sites of free-ranging dogs in urban and semi-urban localities and understand the denning preferences of pregnant females. We hypothesize that dogs would prefer denning sites that provide greater protection from predators, less anthropogenic disturbance and easy access to resources.

## Results

### Den characteristics

**Figure 1:**
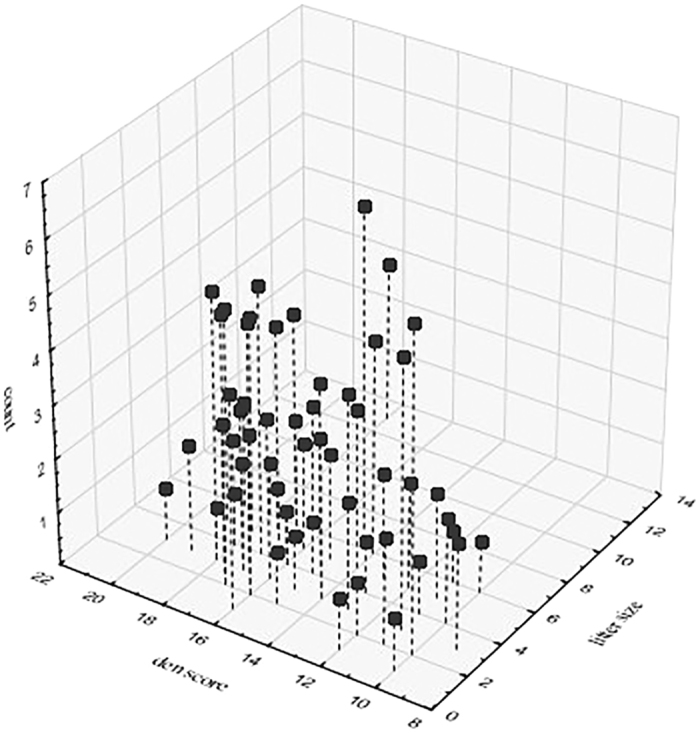
A 3d scatterplot showing the distribution of den scores for various observed litter sizes (N = 148 dens).

Dens were seasonal, most having been located between October and March. Dens could be highly protected and sheltered (inside buildings or other structures), or quite open (located in fields and by the sides of roads). There was no apparent preference of the mothers for sheltered dens (chi-square test: χ^2^ = 0.014, df = 1, p < 0.907). Pregnant females preferred den sites that gave them access to human provided food (begging), rather than direct scavenging at dustbins and garbage dumping sites (chi-square test: χ^2^ = 44.938, df = 1, p < 0.0001). Den scores ranged from 9 to 20 (normalized scores 6.25 to 75), with the highest percentage of dens (14.19%) having scores 17 and 18 (normalized scores 56.25 and 62.5) (Please see Supplementary Information 1 for more details). Average litter size was 4.07 ± 1.99. The den score was not correlated with the size of the litter born in the respective dens (linear regression analysis: R^2^ = 0.003, F = 0.472, p = 0.493), suggesting that the mothers might not be choosing dens based on the expected litter size. However, a 3d plot of frequencies against den scores and litter sizes revealed a pattern of higher incidence of medium to large den scores over small ones, irrespective of litter size (Fig. 1), suggesting that there indeed might be a preference for certain parameters that we used to characterize the dens.

**Figure 2:**
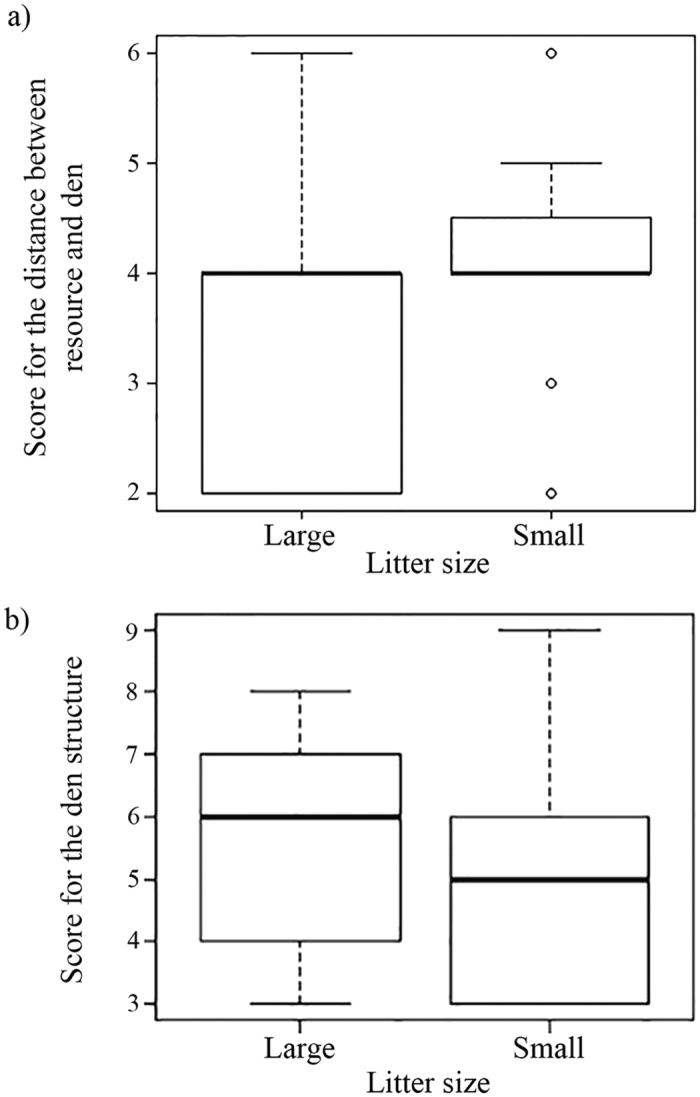
(**a**) Box-whisker plot showing the distance from the den to resources for large (>4 pups) and small (1–4 pups) litters. (**b**) Box-whisker plot showing the den structure for large (>4 pups) and small (1–4 pups) litters.

The GLMM analysis revealed that the distance of the den from resources like food and water plays a very important role (p = 0.00952) in den selection by the mother (Table 1, Supplementary Information 2). Females that produce small litters tend to select dens that are at an intermediate distance from the resources. On the other hand, females that produce large litters seem to be more flexible in their choice with respect to the distance from resources (Fig. 2a). The structure of the den plays a marginally important role (p = 0.04696) in den selection (Fig. 2b), but the combination of structure and resources is more important (p = 0.01794). Hence it appears that mothers with small litters prefer to be not too close to resources, and generally have smaller dens than mothers with large litters. The survivorship analysis revealed that none of the den characteristics affected pup survival (Supplementary Information 3).

### Den selection

**Figure 3:**
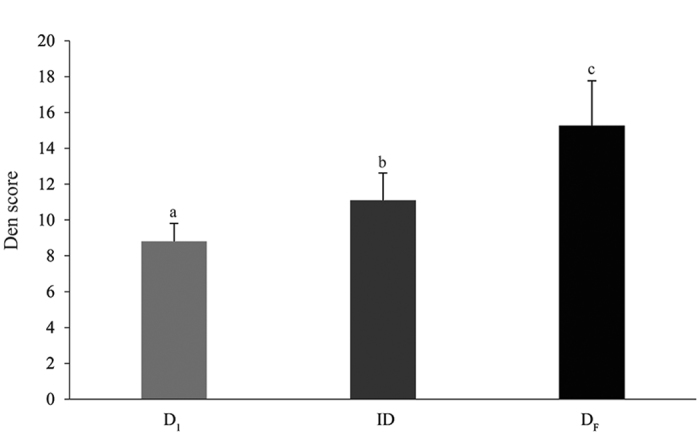
A bar graph showing the mean ± standard deviation of den scores for 15 pregnant females that were tracked for a month prior to whelping. The light gray bar represents the first dens (D_1_), dark gray bar represents the intermediate dens (ID) and the black bar represents the final dens (D_F_).

All the 20 pregnant females that were tracked prior to whelping used at least one den site prior to the final den in which the pups were born. 15 of these females could be tracked for a month prior to whelping, and they occupied an average of 3.6 ± 0.98 dens during this period. The fifteen pregnant females that were tracked for a month had 2–6 intermediate dens, which they occupied for varied times, ranging from 4 to 21 days during the one month that they were tracked. There was significant variation between the first den score (D1S), average intermediate den score (IDS) and final den scores (DFS) for the 15 females (Kruskal-Wallis test, F = 55.925, df = 2, 41, p < 0.0001); wth D1S < IDS < DFS (Wilcoxon paired-sample test with Bonferroni correction, D1-DF: T = 0.00, N = 15, p < 0.0001; D1-ID: T = 0.00, N = 15, p < 0.0001; ID-DF: T = 1.00, N = 15, p < 0.0001; Fig. 3). The number of intermediate dens used by the mother did not depend on the time available to her before whelping (Linear regression: R^2^ = 0.196, F = 3.161, p = 0.099), suggesting that the mothers actively selected dens, settling in one that they preferred, irrespective of the number of den sites they sampled.

The pregnant females covered large distances in search of dens every day (mean ± s.d. = 312.476 ± 182.034 m). The den score difference between the final and first dens (D_1_D_F_) increased with the linear distance between the dens (Linear regression: R^2^ = 0.456, F = 10.908, p = 0.006). D_1_D_F_, however, did not increase with the total distance covered over the one month period of observations, considering the actual paths taken by the females (Linear regression: R^2^ = 0.130, F = 1.938, p = 0.187), but increased with the total number of days spent in intermediate dens (Linear regression: R^2^ = 0.314, F = 5.944, p = 0.030). The linear distance between the first and final dens increased with an increase in the distance covered in searching (Linear regression: R^2^ = 0.316, F = 5.998, p = 0.029). Thus, with increased searching effort the pregnant females were more likely to find more suitable dens, and they indeed searched actively, settling for the most comfortable den available. Majority of final dens had a score of 17 or 18, for both the pregnant females’ data set and the population level data (Supplementary Information 4, Table S2), suggesting 17–18 to be the optimal den score (Supplementary Information 4, Fig. S1).

## Discussion

Free-ranging dogs showed considerable variation in the selection of their denning sites, from open fields to highly protected shelters, small and dark holes to large and well-lit spaces. However, in spite of the large distribution of den scores observed, there seemed to be an optimal den score (scores 17 and 18) which was evident not only from the survey of birthing dens from the population, but also from observations of pregnant females. Unlike wolves and coyotes, which prefer to den away from humans^56^,^60^,^67^,^68^, even while exploiting resources in human dominated landscapes, free-ranging dogs routinely chose sites of high human activity like markets, railway platforms and residential buildings for dens. Canids typically prefer to den close to water sources^67^,^69^,^70^, and this was also true of the free-ranging dogs. They selected dens close to resources that included water sources as diverse as dripping taps to natural water bodies.

Smaller litters tended to be born at dens that were located at intermediate distances from resources and of relatively smaller size, as compared to the dens of larger litters. Dens with larger litters could be found at variable distances from resources. This could mean that the pregnant females are able to judge whether they would be giving birth to small or large litters. However, it is more likely that the mothers simply select dens in which they are comfortable; being heavier and bigger in size while carrying more pups, they would thus prefer larger dens in spite of their distance from resources, leading to this pattern of den selection, without necessarily involving cognitive abilities of the pregnant females. The pregnant females actively searched for denning sites prior to whelping, and routinely rejected a previously selected den for a new one, with higher final den scores. This trend was so strong that for only one of the 20 females the final den had a lower score than the one preceding it. On average, the mothers settled in dens having higher scores than the first dens as well as the average score of the intermediate dens. The pregnant females actively covered large distances in search of dens, and the chances of finding better dens increased as they ventured further from the first dens. Thus it is possible for pregnant females to move away from their core territories in search of better denning sites, and we have indeed observed several cases of pregnant females moving into unoccupied territories in the past (Bhadra *et al*., pers. obs.). Since neither the searching effort, nor the final den scores were correlated with litter size, it is most likely that the mothers searched actively for preferred dens, settling for the best available site, considering comfort in terms of available space and resources, rather than the expected size of her litter.

Canids like wolves and coyotes that live close to humans, tend to den in secluded places, away from human disturbance, and respond to human disturbance at den sites by shifting their pups^60^,^71^,^72^,^73^. Interestingly, the presence of human disturbance did not affect the choice of den sites in the free-ranging dogs. On the contrary, they preferred humans as food sources over foraging sites like dustbins, i.e., they depended more on begging than scavenging^41^,^43^. Moreover, while the mothers shifted dens prior to whelping, they did not appear to shift their pups at all. The analysis of the mortality levels in the first month of pup age also shows that the mother’s choice of den site does not influence the survival of pups. Ironically, the mortality of pups increases with age, reaching 81% by the seventh month, of which 63% is human induced^66^.

The tendency of the pregnant females to den in the proximity of people seems to be a double edged sword. On the one hand, this provides easy access to resources (through begging), without facing competition from conspecifics, while on the other, this exposes the pups to interfering humans, which can be potentially harmful. The socio-cultural milieu of India promotes tolerance, and ancient texts even recommend regular feeding of scavengers like dogs and crows^74^. Moreover, the laws of the land and animal rights activists ensure that the dogs lead a relatively unthreatened life on streets. Hence, the risks of denning close to humans are by far outweighed by the advantages of this behaviour. Our results show that while dogs have retained the behaviour of den selection prior to whelping, much like their ancestors, they have adapted to the human dominated environment by accepting, rather than avoiding the proximity to humans while denning. Indeed, they seem to be exploiting the tendency of humans to respond to them^75^ for easy access to resources. Free-ranging dogs are capable of hunting in packs like their ancestors^39^,^76^,^77^, the wolves, but in the urban habitat, they thrive as scavengers, living in small social groups and often choosing to forage solitarily^42^,^52^. Denning close to humans enables them to avoid competition with other mesocarnivores and predation. The tendency to den in and around human habitations and beg from humans seems to be unique to dogs, and quite in contrast to other canids. In spite of the high mortality of pups induced by humans, the ability to exploit humans as sources of food and shelter appears to be a behavioural adaptation that helps dogs to survive in the urban landscape.

## Methods

### Den characteristics

Free-ranging dog den sites were identified through surveys^42^ between 2010 and 2015 by tracking dog groups and following pregnant females in various parts of West Bengal, India. In this study, we used 148 den sites in rural (5), urban (90) and semi urban (53) localities. The study was conducted in and around Kolkata (22.5667°N, 88.3667°E), Saltlake (22.5800°N, 88.4200°E), Kalyani (22.9750°N, 88.4344°E), IISER-K campus in Mohanpur (22.9638°N, 88.5246°E), Barasat (22.7200°N, 88.4800°E) and Barrackpore (22.7600°N, 88.3700°E), West Bengal, India. The localities were selected arbitrarily based on convenience and safety of sampling, in residential or business areas or both.

Observers visited the areas at random times during the day and walked on all roads and by-lanes^42^. Whenever a den was sighted, it was measured for length, breadth and height (if applicable), and a record was made of its physical parameters, distance from resources, nature of available resources and the size of the litter in the den (Supplementary Information 1). Each den was photographed and the date of birth of the pups was recorded with inputs from local people whenever possible. The nature of the resources available were recorded as dustbins (when dogs were seen to scavenge from dustbins and other garbage dumping sites) and people (when dogs were seen to obtain food by begging, or were actively fed by humans). We expected the dogs to prefer dens that provided greater protection from predators, had less anthropogenic disturbance and easy access to resources. Based on this, the den characteristics were given scores, and thus each den obtained a total score based on its characteristics (Supplementary Information 1). For a subset of the dens (41 dens out of total 148 dens), we also obtained data on mortality of the pups up to one month of age, because beyond this stage pups do not stay inside the dens any longer.

### Den selection

In order to understand the den selection procedure of expecting mothers, pregnant females were identified and followed until parturition. Each female was observed twice a day, every alternate day, from 0700–1000 h and 1500–1800 h. The study was conducted over three breeding seasons, between February 2013 and March 2015, in Kolkata, Saltlake and Barrackpore. We obtained data on 20 pregnant females, of which 15 females could be tracked for as long as a month before parturition, while the rest whelped earlier. For each pregnant female, we recorded all the above mentioned criteria (other than litter details) for the first den (D_1_), the intermediate dens (ID) which she was seen to occupy, and the final den (D_F_) in which she whelped. We also recorded the date of birth, size of the litter and sex of the pups for the final dens. While following pregnant females, we recorded daily distance travelled and the corresponding route on a map, which was used to estimate the length of the path covered over the entire tracking period and calculate the linear distance between her first and final dens. We also maintained records of the time spent resting in each intermediate den. The distance between the first and final dens, the total path covered in searching and the time spent in intermediate dens were used as estimates of the searching effort of the females for locating preferred den sites. We calculated the intermediate den score (IDS) by averaging over all the den scores from the first to the last but one den occupied by the pregnant females. The difference in scores between the first and final dens was also calculated and designated as D_1_D_F._

### Statistical analysis

All statistical analyses were carried out using StatistiXL 1.10, Statistica version 12 and R statistics (R Studio)^78^. The dens were given scores for each of the characteristics, and the total den score was computed. We used a generalized linear mixed model (GLMM) to investigate the importance of den characteristics for den site. For the GLMM analysis, litter size was considered as the response variable, with den characteristics as the fixed effects while the identity of the groups and the year of observation were taken as the random effects. Den characteristics were grouped into four categories, structure (area, height, and level), resources (distance from food and water), presence of human disturbances and den quality (nature of shade and light availability) (Supplementary Information 2). Human disturbances were quantified based on the qualitative data of human (especially child) interactions with the new born pups. Many cases were observed where human child took away the pups as pet^66^ or interrupted the suckling bouts or sometimes beaten the pups or the mother as a part of their play. Since there were very few dens with large litters (having 7 or more pups) and mean litter size was 4, we grouped the litter sizes into two categories, small (1–4) and large (>4), and ran the GLMM analysis for a binomial distribution of litter size. In order to check if the dens selected by the mothers impact the survival of the pups (from birth to one month of pup age), we ran a Cox mixed-effects model for survivorship, considering den characteristics (structure, resources, human disturbances and quality) as fixed effects. Age of pups and survival up to one month of age (survived or not survived) were the response variables. Group identity and year of observation were added as random effects (Supplementary Information 3).

We used a Kruskal-Wallis test to compare the first den score, average intermediate den score and final den score. The three sets of den score were then compared using a Wilcoxon paired-sample test, in order to check if the females chose “better” dens while rejecting “poorer” ones. Linear regression analysis was performed to check if the number of intermediate dens used by the pregnant females depended on the time available to them before whelping.

In order to understand if the mother’s searching effort led to her finding better dens, we carried out linear regression analyses of the difference in scores between the final and first dens (D_1_D_F_) with the following: (i) the linear distance between the first and final dens; (ii) the total distance covered over the one month period of observations, considering the actual paths taken by the females and (iii) the total number of days spent in intermediate dens. Another linear regression analysis was used to check if the distance between the first and final dens increased with the total path covered in den search.

### Ethical statement

No dogs were harmed during this study and all work that has been reported here was purely observation based. The methods reported in this paper were approved by the animal ethics committee of IISER Kolkata (approval number: 1385/ac/10/CPCSEA), and in accordance with approved guidelines of animal rights regulations of the Government of India.

## Additional Information

**How to cite this article**: Majumder, S. S. *et al*. Denning habits of free-ranging dogs reveal preference for human proximity. *Sci. Rep.* **6**, 32014; doi: 10.1038/srep32014 (2016).

## Supplementary Material

Supplementary Information

## Figures and Tables

**Table 1:** Results of the GLMM analysis considering a Binomial distribution for litter size.

Fixed effects				
	Estimate	Std. Error	Z value	Pr(>|z|)
(Intercept)	5.2796	2.5443	2.075	0.03798*
Structure	−0.8409	0.4233	−1.987	0.04696*
Distance from resource	−1.7247	0.6652	−2.593	0.00952**
Structure* Distance from resource	0.2508	0.1060	2.367	0.01794*
**Random Effects**				
**Groups**	**Name**	**Variance**	**Std.Dev.**	
Individual ID	(Intercept)	0.01231	0.1109	
Year of observation	(Intercept)	0.00000	0.0000	
