# Supplementary material for: Denning habits of free-ranging dogs reveal preference for human proximity
Source: Sci Rep. 2016 Aug 18;6:32014. doi: 10.1038/srep32014 (PMC4989282; doi:10.1038/srep32014)
Supplement: Supplementary Information [file srep32014-s1.pdf]

Denning habits of free-ranging dogs reveal preference for human proximity

Sreejani Sen Majumder[1], Manabi Paul[1], Shubhra Sau[1] and Anindita Bhadra[1*]

[1] Department of Biological Sciences,

Indian Institute of Science Education and Research Kolkata, India

[*]**Address for Correspondence:**

Behaviour and Ecology Lab, Department of Biological Sciences,

Indian Institute of Science Education and Research Kolkata

Mohanpur Campus, Mohanpur,

PIN 741246, West Bengal, INDIA

tel. 91-33-66340000-1232

fax +91-33-25873020

e-mail: abhadra@iiserkol.ac.in

Supplementary Information 1

**Detailed characterization**		
**Parameters**	**Features**	**Score**
Level of dens	Under ground<br>Above ground<br>Ground	3<br>2<br>1
Shade	Full shade<br>Moderate shade<br>Without shade	3<br>2<br>1
Light	Bright light<br>Moderate light<br>Dark	3<br>2<br>1
Human disturbance	Highly disturbed<br>Moderate<br>Without disturbance	1<br>2<br>3
Distance from water source	2-10 meter<br>< 2 meter<br>> 10 meter	3<br>2<br>1
Distance from food source	2-10 meter<br>< 2 meter<br>> 10 meter	3<br>2<br>1
Area	> 1 meter square<br>.5-1 meter square<br>< 0.5 meter square	3<br>2<br>1
Height	>1 meter<br>< 1 meter<br>No height	3<br>2<br>1
**Composite Characterization**		
**Structure of den**	Level+Height+Area	
**Quality of den**	Shade+Light+Disturbance	
**Resources**	Distance from food source + Distance from water source	

Table S1: Each den was first characterized for 8 parameters, as per the scoring method shown here. The parameters were then pooled into composite characteristics for each den. The highest possible den score was 24, and the lowest 8. This score was then scaled from 0 to 100 for each den using the formula: 100 * {(DS-8)/ (24-8)}, thus obtaining the normalized den score.

Supplementary Information 2

We have sorted the litter size into two categories i.e. small and large (mothers having a litter of 1-4 pups are considered as having small litter and if a mother has more than 4 pups in her litter then the litter size is considered as large). In that way for each mother either she has a small litter or a large litter. We have made a model considering the distribution as Binomial. We have used the "lme4" package in "R studio" for the Generalized linear mixed effect model (GLMM) analysis.

Variables

Response variable

Litter size (either small or large): **litsize**

Fixed effects

Structure of den: **str**

Distance of food source from the den: **res**

Human disturbances: **dis**

Quality of den (light and shade): **qul**

Random effects

Group identity: **ind**

Year of data collection: **yr**

The results are:

Model 1: *litsize ~ str + res + dis + qul + (1 | ind) + (1 | yr)*

Control: glmerControl(optimizer = "bobyqa")

Random effects:

Groups	Name	Variance	Std.Dev.
ind	(Intercept)	0.01743	0.132
yr	(Intercept)	0.00000	0.000

Number of obs: 148, groups: ind, 148; yr, 5

Fixed effects:

	Estimate	Std. Error	z value	Pr(>|z|)
(Intercept)	-0.515679	1.077319	-0.479	0.632
str	0.146432	0.109836	1.333	0.182
res	-0.240395	0.156305	-1.538	0.124
dis	0.024109	0.214348	0.112	0.910
qul	-0.001998	0.118575	-0.017	0.987

Model 2: *litsize ~ str + res + dis + (1 | ind) + (1 | yr)*

Control: glmerControl(optimizer = "bobyqa")

Random effects:

Groups	Name	Variance	Std.Dev.
ind	(Intercept)	0.01738	0.1319
yr	(Intercept)	0.00000	0.0000

Number of obs: 148, groups: ind, 148; yr, 5

Fixed effects:

	Estimate	Std. Error	z value	Pr(>|z|)

(Intercept)	-0.52319	0.98273	-0.532	0.594
str	0.14589	0.10496	1.390	0.165
res	-0.23984	0.15291	-1.569	0.117
dis	0.02413	0.21435	0.113	0.910

Model 3: *litsize ~ str + res + (1 | ind) + (1 | yr)*

Control: glmerControl(optimizer = "bobyqa")

Random effects:

Groups	Name	Variance	Std.Dev.
ind	(Intercept)	0.01801	0.1342
yr	(Intercept)	0.00000	0.0000

Number of obs: 148, groups: ind, 148; yr, 5

Fixed effects:

	Estimate	Std. Error	z value	Pr(>|z|)
(Intercept)	-0.4543	0.7680	-0.592	0.5541
str	0.1463	0.1049	1.394	0.1632
res	-0.2443	0.1479	-1.652	0.0986 .

Model 4*: litsize ~ str * res + (1 | ind) + (1 | yr)*

Control: glmerControl(optimizer = "bobyqa")

Random effects:

Groups	Name	Variance	Std.Dev.
ind	(Intercept)	0.01231	0.1109
yr	(Intercept)	0.00000	0.0000

Number of obs: 148, groups: ind, 148; yr, 5

Fixed effects:

	Estimate	Std. Error	z value	Pr(>|z|)
(Intercept)	5.2796	2.5443	2.075	0.03798 *
str	-0.8409	0.4233	-1.987	0.04696 *
res	-1.7247	0.6652	-2.593	0.00952 **
str:res	0.2508	0.1060	2.367	0.01794 *

Supplementary Information 3

We have used the “Coxme” package in “R studio” for pups’ survival analysis.

Variables

Response variables

Age of the pups: **age**

Survival of the pups up to one month of their age: **earlysrv**

Fixed effects

Structure of den: **str**

Quality of den (light and shade): **qul**

Human disturbances: **dis**

Distance of food source from the den: **res**

Random effects

Group identity: **ind**

Year of data collection: **yr**

The results are:

Cox mixed-effects model fit by maximum likelihood

Model: *Surv(age, earlysrv) ~ res + str + qul + dis + (1 | ind/yr)*

Fixed coefficients

	coef	exp(coef)	se(coef)	z	p
res	0.37298878	1.452068	0.2336843	1.60	0.11
str	0.06877629	1.071197	0.1953613	0.35	0.72
qul	0.17221203	1.187930	0.2066397	0.83	0.40
dis	0.38312792	1.466866	0.3377779	1.13	0.26

Random effects

Group	Variable	Std Dev	Variance
find/fyr	(Intercept)	0.4001344	0.1601075
find	(Intercept)	0.4001905	0.1601524

Supplementary Information 4

SL no.	Habitat	Litter size	Distance from		Disturbance	Level	Height	Shade	Light	Area	Score	
			Food	Water							Total	Max
1	Semi	3	1	1	3	2	3	3	3	1	17	24
2	Urban	6	1	1	3	1	3	3	3	2	17	24
3	Urban	2	1	1	3	1	3	3	3	3	18	24
4	Semi	2	1	1	3	2	2	3	2	3	17	24
5	Urban	2	1	1	3	1	2	3	3	3	17	24
6	Semi	5	1	1	3	3	1	3	3	2	17	24
7	Urban	6	1	1	3	1	3	3	3	2	17	24
8	Urban	2	1	1	3	1	2	3	3	3	17	24
9	Urban	4	1	**2**	3	1	2	3	2	3	17	24
10	Urban	7	1	**2**	3	2	2	3	2	3	18	24
11	Urban	5	1	**2**	3	1	2	3	3	3	18	24
12	Urban	4	1	**2**	3	1	2	3	2	3	17	24
13	Urban	6	1	3	1	1	3	3	2	3	17	24
14	Urban	4	1	3	2	1	3	3	3	2	18	24
15	Semi	4	1	3	3	2	3	3	2	1	18	24
16	Rural	6	**2**	1	3	1	3	3	1	3	17	24
17	Semi	1	**2**	1	3	2	1	3	3	2	17	24
18	Urban	3	**2**	**2**	1	2	3	3	2	3	18	24
19	Semi	5	**2**	**2**	1	1	3	3	3	3	18	24
20	Urban	14	**2**	**2**	3	2	3	3	1	1	17	24
21	Rural	5	**2**	**2**	3	1	3	3	1	3	18	24
22	Urban	3	**2**	**2**	3	2	2	3	2	2	18	24
23	Urban	8	**2**	**2**	3	2	2	3	1	3	18	24
24	Urban	2	**2**	**2**	3	1	1	3	3	3	18	24

25	Urban	8	**2**	**2**	3	2	3	2	1	3	18	24
26	Semi	4	**2**	**2**	3	2	3	1	1	3	17	24
27	Urban	2	**2**	**2**	3	1	1	3	3	3	18	24
28	Urban	5	**2**	**2**	3	1	3	3	1	3	18	24
29	Urban	2	**2**	3	2	1	2	3	3	1	17	24
30	Urban	1	**2**	3	2	1	2	3	3	1	17	24
31	Name	4	**2**	3	2	1	2	3	3	2	18	24
32	Urban	5	**2**	3	2	1	3	2	2	3	18	24
33	Urban	4	**2**	3	3	2	3	2	1	2	18	24
34	Semi	2	3	1	1	2	3	3	3	1	17	24
35	Semi	3	3	1	2	2	3	3	3	1	18	24
36	Urban	5	3	**2**	1	1	3	3	1	3	17	24
37	Semi	5	3	**2**	1	3	1	3	1	3	17	24
38	Urban	3	3	**2**	3	1	3	3	1	1	17	24
39	Urban	4	3	**2**	3	1	2	3	2	2	18	24
40	Semi	2	3	**2**	3	1	1	3	3	1	17	24
41	Semi	2	3	3	1	1	3	2	2	3	18	24
42	Semi	3	3	3	1	3	3	1	2	1	17	24
43	Urban	3	3	3	2	1	3	1	1	3	17	24
44	Urban	2	3	3	3	2	3	1	1	2	18	24
45	Urban	2	3	3	3	1	3	1	1	3	18	24
46	Urban	2	3	3	3	2	1	1	1	3	17	24
47	Urban	7	3	3	3	3	1	1	1	3	18	24

Table S2: This table summarizes the scores for the various characteristics that were obtained by the 47 dens (42 from the population l evel sample and 5 from the pregnant females' sampling), that had a total score of 17 or 18.

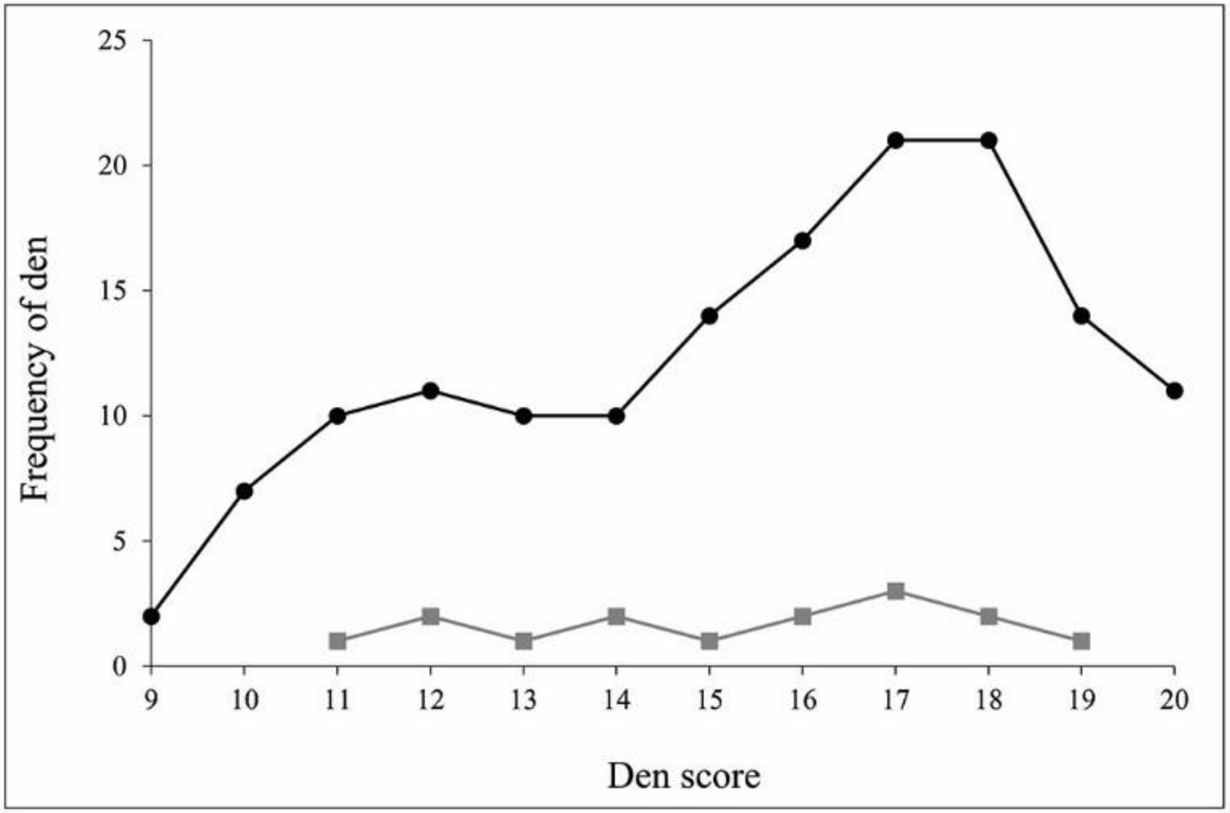

Figure S1: A plot showing the frequencies of dens with various den scores. The black dots represent the population level sample and the gray dots represent the data for the final dens of the 20 pregnant females.
